# Cooperation of SRPK2, Numb and p53 in the malignant biology and chemosensitivity of colorectal cancer

**DOI:** 10.1042/BSR20191488

**Published:** 2020-01-17

**Authors:** Guosen Wang, Weiwei Sheng, Jingtong Tang, Xin Li, Jianping Zhou, Ming Dong

**Affiliations:** 1 Department of General Surgery, The First Affliated Hospital, Nanchang University, Nanchang 330006, Jiangxi, China; 2 Department of Gastrointestinal Surgery, The First Hospital, China Medical University, Shenyang 110001, Liaoning, China

**Keywords:** chemosensitivity, colorectal cancer, malignant biology, Numb, p53, SRPK2

## Abstract

Serine-arginine protein kinase 2 (SRPK2) is aberrantly expressed in human malignancies including colorectal cancer (CRC). However, little is known about the molecular mechanisms, and the role of SRPK2 in chemosensitivity remains unexplored in CRC. We recently showed that SRPK2 promotes pancreatic cancer progression by down-regulating Numb and p53. Therefore, we investigated the cooperation between SRPK2, Numb and p53 in the cell migration, invasion and chemosensitivity of CRC *in vitro*. Here, we showed that SRPK2 expression was higher in CRC tumors than in nontumor tissues. SRPK2 expression was positively associated with clinicopathological characteristics of CRC patients, including tumor differentiation, T stage, N stage and UICC stage. Additionally, SRPK2 had no association with mutant p53 (mtp53) in SW480 and SW620 cells, but negatively regulated Numb and wild-type p53 (wtp53) in response to 5-fluorouracil or cisplatin treatment in HCT116 cells. Moreover, SRPK2, Numb and p53 coimmunoprecipitated into a triple complex with or without the treatment of 5-fluorouracil in HCT116 cells, and p53 knockdown reversed the up-regulation of wtp53 induced by SRPK2 silencing with chemical agent treatment. Furthermore, overexpression of SRPK2 increased cell migration and invasion and decreased chemosensitivity to 5-fluorouracil or cisplatin in HCT116 cells. Conversely, SRPK2 silencing decreased cell migration and invasion and increased chemosensitivity to 5-fluorouracil or cisplatin, yet these effects could be reversed by p53 knockdown under chemical agent treatment. These results thus reveal a novel role of SRPK2-Numb-p53 signaling in the progression of CRC and demonstrate that SRPK2 is a potential therapeutic target for CRC clinical therapy.

## Introduction

Colorectal cancer (CRC) is one of the most common malignancies worldwide [1,2], with an estimated incidence rate of 6.1% and mortality rate of 9.2% in 2018 [3]. Strong invasion, metastasis and chemoresistance contribute to the dismal prognosis of CRC patients. Thus, identification of the potential molecular mechanisms underlying the malignant biology and combating the drug resistance of CRC is urgently needed.

Serine-arginine protein kinase 2 (SRPK2) is a protein kinase that was cloned in 1998, based on its ability to phosphorylate serine/arginine (SR) proteins [4,5]. Several reports have suggested a contributory role of SRPK2 in the pathogenesis of neurodegenerative diseases [6,7], the infectivity of multiple viruses [8,9] and the posttranscriptional regulation of lipogenesis [10]. In addition, SRPK2 is overexpressed in several cancer types, including leukemia and lung, colon, prostate and pancreatic cancers [11–15]. Wang et al. [13] showed that SRPK2 promoted the growth and migration of colon cancer cells. Nevertheless, the molecular mechanisms by which SRPK2 regulates malignant biology in CRC are still poorly understood. Additionally, how SRPK2 mediates chemosensitivity and the association between SRPK2 expression and clinical data remain unexplored in CRC. Most recently [15], we reported that SRPK2 promoted cell migration and invasion, and decreased chemosensitivity to gemcitabine or oxaliplatin treatment via the Numb and p53 signaling pathway in pancreatic cancer.

However, the crucial roles of the association between SRPK2, Numb and p53 in CRC remain elusive; these roles are assessed in the present study.

## Materials and methods

### Patient samples and cell lines

CRC tissues were obtained from patients at the First Hospital of China Medical University from 2015 to 2017, with approval from the Institutional Review Board of the China Medical University. All patients provided written informed consent. Pathological diagnoses were performed by two pathologists independently.

The mutant p53 (mtp53) SW480 and SW620 cell lines were purchased from the Cell Bank of the Chinese Academy of Sciences (Shanghai, China), as well as the wild-type p53 (wtp53) HCT116 cell line. Cells were cultured in RPMI-1640 medium (HyClone, U.S.A.) supplemented with 10% fetal bovine serum (FBS, HyClone, U.S.A.) at 37°C in a humidified incubator with 5% CO_2_.

### Immunohistochemistry analysis

Immunohistochemistry was performed on 4 μm consecutive sections from paraffin-embedded specimens by using the peroxidase protocol. The UltraSensitive™ SP IHC Kit (MXB Bio, China) was used according to the manufacturer’s instructions. A primary antibody against SRPK2 (Abcam, U.K., 1:400) was applied. The signals were developed using a DAB kit (MXB Bio, China), and then mounted for microscopy.

Immunostainings were evaluated by two blind-folded pathologists according to the protocol by Masunaga et al. [16]. Staining intensity was evaluated as 0 (negative), 1 (mild), 2 (medium) and 3 (intense). The extent of staining was recorded in four grades: 0 (0%), 1 (1–25%), 2 (26–50%), 3 (51–75%) and 4 (76–100%). A final score was established by adding the staining intensity and extent (0–7). Positive expression was considered when the final staining score was >2.

### Quantitative real-time reverse-transcription polymerase chain reaction (qRT-PCR) analysis

Total RNA was isolated from CRC tissues using TRIzol reagent (Takara Bio, Japan) according to the manufacturer’s protocol. cDNA was synthesized from the isolated total RNA using the Expand Reverse Transcriptase Kit (Takara Bio, Japan) and subjected to qRT-PCR analyses using the standard program. The primers used in the present study were SRPK2, 5′-GGAGATAGAAGAATTGGAGCGAGAAGC-3′ (forward) and 5′-CCTCAGCCGCCTCCTCTAATCC-3′ (reverse); and GADPH, 5′-CATGAGAAGTATGACAACAGCCT-3′ (forward) and 5′-AGTCCTTCCACGATACCAAAGT-3′ (reverse). The relative mRNA level was quantified using the ΔΔ-*C*t method.

### Western blotting and immunoprecipitation

Western blotting was performed according to a standard protocol. Briefly, the samples harvested from CRC tissues or cells were separated on SDS-polyacrylamide gels and blotted onto PVDF membranes (Millipore, U.S.A.). After incubating with the primary SRPK2 (Abcam, U.K., 1:1000), Numb (Abcam, U.K., 1:1000), p53 (Proteintech, U.S.A., 1:1000) or GAPDH (Proteintech, U.S.A., 1:3000) antibodies overnight at 4°C, the blots were subsequently probed with HRP-conjugated secondary antibody (Santa Cruz, U.K., 1:20000). Images were captured with the ECL detection kit (Thermo Scientific, U.S.A.).

Immunoprecipitation was performed as described in our previous study [15], with the primary antibodies SRPK2 (Abcam, U.K., 1:80), Numb (Abcam, U.K. 1:80), p53 (Proteintech, U.S.A., 1:100) or control IgG (Santa Cruz, U.K., 1:100).

### Generation of stable cell lines and transient transfection for rescue experiment

The generation of stable cell lines was carried out as previously described [15]. Lentiviruses were produced by Genechem (Genechem Co, China). SRPK2-silencing stable cell lines were constructed using the SRPK2-sgRNA (sg-SRPK2) and sgRNA control (Scramble) lentiviruses, while SRPK2 overexpressing were constructed using the GV358-SRPK2-GFP plasmid (SRPK2-GFP) and corresponding empty plasmid (GFP). Puromycin (Sigma, U.S.A.) was used to screen the transfected cells.

The siRNA transient transfection for rescue experiment was performed using Lipofectamine 3000 reagent (Invitrogen, U.S.A.) in accordance with the manufacturer’s protocol. All siRNAs were purchased from GenePharma Company (GenePharma Co, China). The sense sequences were as follows: p53 siRNA, 5′-CUACUUCCUGAAAACAACGTT-3′; and siRNA control, 5′-UUCUCCGAACGUGUCACGUTT-3′. Transfection efficiency was routinely verified by Western blot analysis.

### Cell invasion and migration assays

Matrigel-coated and -uncoated modified Boyden chambers (BD Biosciences, U.S.A.) were used to evaluate cell invasion and migration. Briefly, transfected cells suspended in FBS-free growth medium were plated into the upper chamber, and growth medium containing 10% FBS was loaded in the lower chamber as an attractant. After 24-h incubation, cells were fixed in 4% paraformaldehyde and stained with 0.1% Crystal Violet (Sigma, U.S.A.). The cells on the upper chamber, which had invaded or migrated to the lower chamber, were removed with a cotton swab and were counted by using a microscope (Nikon Microphot-FX, Japan) in five randomly selected field at ×20 magnification.

### CCK-8 cell chemosensitivity assay

Cell chemosensitivity was measured using the CCK-8 assay. Briefly, transfected cells were seeded in 96-well plates and treated with a concentration gradient of 5-fluorouracil (Abcam, U.K.) or cisplatin (Abcam, U.K.) for 48 h. Then, each well was incubated with 10 μl CCK-8 (Dojindo, Japan) for 2 h at 37°C. The optical density (OD) value was read at 450 nm. Data are presented as the percentage of treated cells compared with that of control cells.

### Statistical analyses

All data analyses were carried out with SPSS 13.0 software. Paired sample *t*-tests were used to evaluate the statistical significance of SRPK2 expression in tumors and corresponding nontumor tissues. The relationship between SRPK2 expression and clinical features was assessed by χ^2^ tests. Differences in the cell migration, invasion and chemosensitivity assays were estimated using Student’s *t*-tests. Data from three independent experiments were expressed as the mean ± SD. *P* < 0.05 was considered statistically significant.

## Results

### SRPK2 was more highly expressed in CRC tumors compared with adjacent nontumor tissues

**Figure 1:**
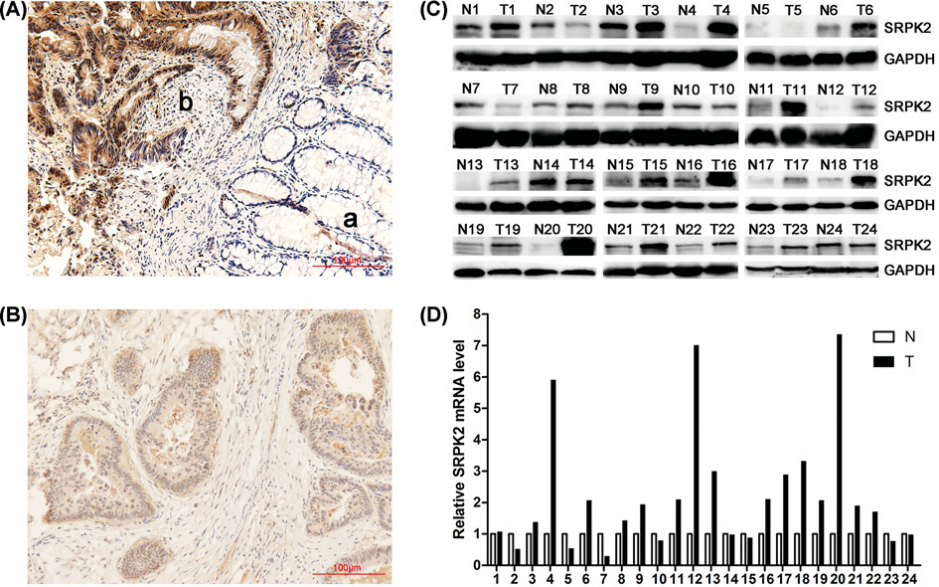
SRPK2 expression is elevated in CRC tumors compared with nontumor tissues (**A** and **B**) Representative images of SRPK2 protein expression in nontumor tissues (Aa), moderately differentiated tumor tissues (Ab) and highly differentiated tumor tissues (B) of 111 CRC cases, as detected by immunohistochemistry at ×200 magnification. (**C** and **D**) SRPK2 protein (C) and mRNA (D) levels in tumor tissues (T) and adjacent nontumor tissues (N) of 24 CRC cases, as detected by Western blotting and qRT-PCR respectively.

We first assessed SRPK2 protein expression by immunohistochemistry in 111 CRC cases. SRPK2 expression in tumors was significantly up-regulated compared with matched adjacent nontumor tissues (61.3%, 68/111 vs 36.9%, 41/111; *t* = 3.72, *P* < 0.001) (Figure 1A,B). Next, we examined SRPK2 protein and mRNA expression by Western blotting and qRT-PCR in 24 CRC cases, again finding elevated expression in tumors compared with matched adjacent nontumor tissues (*t* = 3.631, *P* = 0.001; *t* = 3.021, *P* = 0.006) (Figure 1C,D).

### Association of SRPK2 expression with clinical features

The association between SRPK2 expression and clinical features was examined in 111 CRC cases. As shown, SRPK2 expression was positively associated with tumor differentiation (*P* = 0.019), as well as the T (*P* = 0.018), N (*P* < 0.001) and UICC (*P* < 0.001) classifications (Table 1).

**Table 1:** Association of SRPK2 expression with clinical features in CRC patients

Parameters	No. of patients	SRPK2		*P*
		Negative	Positive	
Cases	111	43	68	
Age (years)				
≤60	42	15	27	0.690
>60	69	28	41	
Gender				
Male	57	23	34	0.846
Female	54	20	34	
Tumor location				
Colon	52	19	33	0.699
Rectum	59	24	35	
Tumor size (cm)				
<5.0	67	29	38	0.240
≥5.0	44	14	30	
Differentiation				
Well	25	15	10	0.019
Moderate and poor	86	28	58	
T stage				
T1+T2	23	14	9	0.018
T3+T4	88	29	59	
N stage				
N0 (negative)	71	39	32	<0.001
N1 (positive)	40	4	36	
Metastasis				
M0 (negative)	101	42	59	0.106
M1 (positive)	10	1	9	
UICC stage				
I+II	53	34	19	<0.001
III+IV	58	9	49	

### The relationship between SRPK2 and p53 in mtp53 and wtp53 CRC cell lines

**Figure 2:**
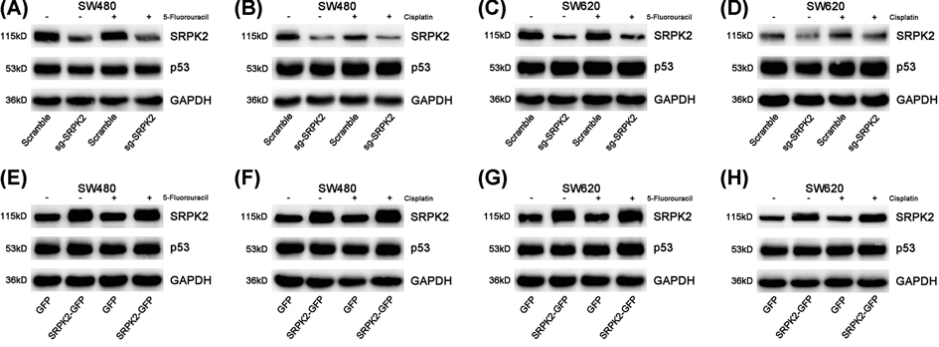
The association of SRPK2 and p53 in mtp53 CRC cell lines (**A** and **B**) SRPK2 silencing had no effect on p53 protein levels in mtp53 SW480 cells, regardless of treatment with 5-fluorouracil (A) or cisplatin (B). (**C** and **D**) SRPK2 silencing had no effect on p53 protein levels in mtp53 SW620 cells, regardless of treatment with 5-fluorouracil (C) or cisplatin (D). (**E** and **F**) SRPK2 overexpression had no effect on p53 protein levels in mtp53 SW480 cells, regardless of treatment with 5-fluorouracil (E) or cisplatin (F). (**G** and **H**) SRPK2 overexpression had no effect on p53 protein levels in mtp53 SW620 cells, regardless of treatment with 5-fluorouracil (G) or cisplatin (H). Statistical significance was determined by Student’s *t*-test.

**Figure 3:**
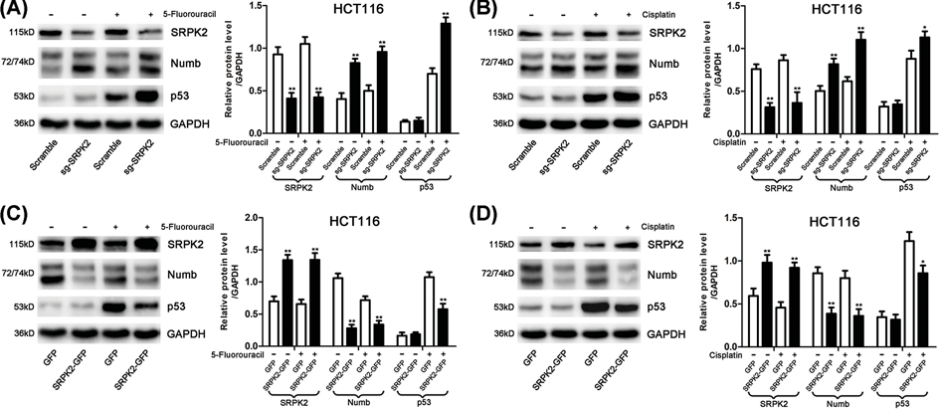
SRPK2 negatively regulates Numb and p53 in wtp53 CRC cells under chemical agent treatment (**A** and **B**) The association of these 3 proteins in SRPK2-silenced HCT116 cells, with or without 5-fluorouracil (A) or cisplatin (B) treatment. (**C** and **D**) The association of these three proteins in SRPK2-overexpressing HCT116 cells with or without 5-fluorouracil (C) or cisplatin (D) treatment. Data are shown as the mean ± SD. Statistical significance was determined by Student’s *t*-test; *P < 0.05, **P < 0.01.

SRPK2-silencing and -overexpressing stable cell lines were successfully constructed (Figures 2 and 3). In SW480 and SW620 cell lines (Figure 2), SRPK2 silencing or overexpression had no effect on mtp53 protein level, and mtp53 expression was unchanged under 5-fluorouracil or cisplatin treatment. In HCT116 cells (Figure 3), the wtp53 protein level did not change in the absence of chemical agent treatment. However, under chemical agent treatment (IC50 for 24 h), wtp53 was activated, and wtp53 protein levels were significantly higher in the sg-SRPK2 group compared with the Scramble group and conversely reduced in the SRPK2-GFP group compared with the GFP group. Moreover, the Numb expression was significantly increased in the SRPK2-silenced cells and conversely decreased in the SRPK2-overexpressing cells, regardless of treatment with chemical agents at their IC50 doses. Together, these findings suggested that SRPK2 had no relationship with mtp53 but negatively regulated Numb and wtp53 under chemical agent treatment.

**Figure 4:**
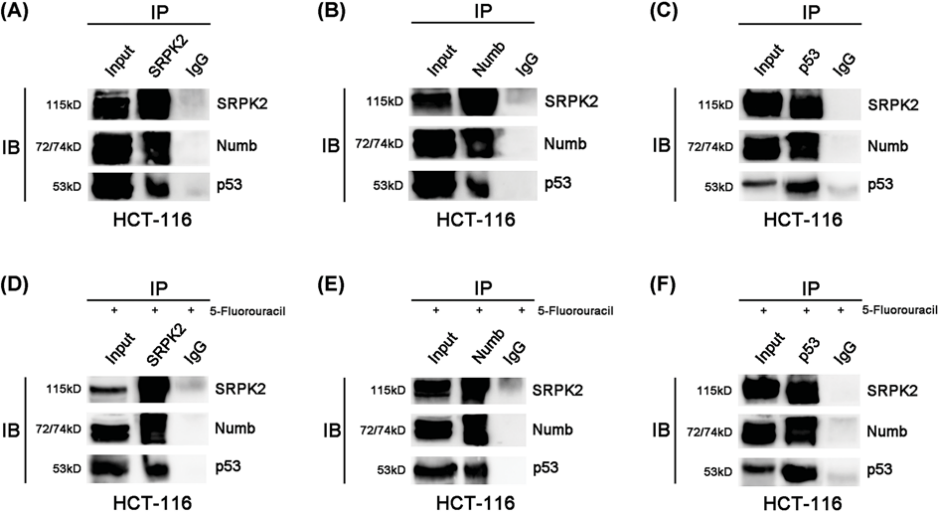
Immunoprecipitation analysis in wtp53 CRC cells without or with 5-fluorouracil treatment (**A–C**) SRPK2 coimmunoprecipitated with Numb and p53 in HCT116 cells without 5-fluorouracil treatment, regardless of whether the SRPK2 (A), Numb (B) or p53 (C) antibody was used. (**D–F**) SRPK2 coimmunoprecipitated with Numb and p53 in HCT116 cells with 5-fluorouracil treatment, regardless of whether the SRPK2 (D), Numb (E) or p53 (F) antibody was used. Input: positive control, IgG: negative control.

Furthermore, immunoprecipitation analysis indicated that SRPK2 could coimmunoprecipitate with Numb and wtp53 in HCT116 cells, regardless of treatment with 5-fluorouracil at its IC50 (Figure 4). These findings suggested a close relationship between these three proteins in wtp53 CRC cells.

### SRPK2 regulated cell migration, invasion and chemosensitivity through the wtp53 signaling pathway

**Figure 5:**
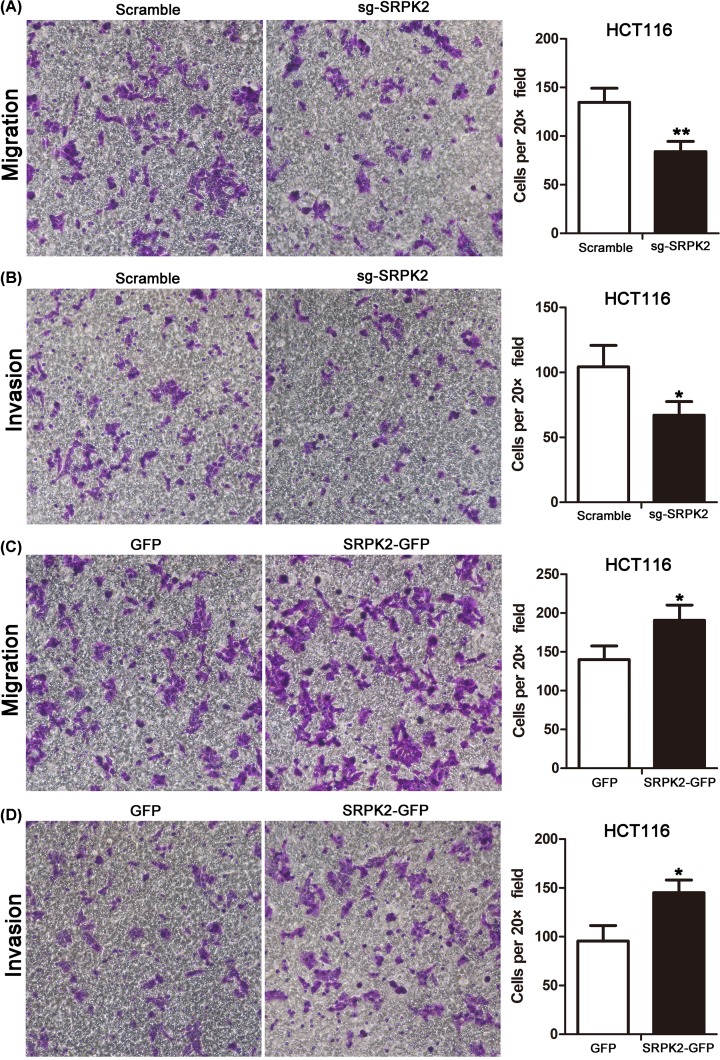
SRPK2 promotes the migration and invasion of wtp53 CRC cells (**A** and **B**) Cell migration (A) and invasion (B) assays in SRPK2-silenced HCT116 cells. (**C** and **D**) Cell migration (C) and invasion (D) assays in SRPK2-overexpressing HCT116 cells. Original magnification ×200. Data are shown as the mean ± SD. Statistical significance was determined by Student’s *t*-test. *P < 0.05, **P < 0.01.

**Figure 6:**
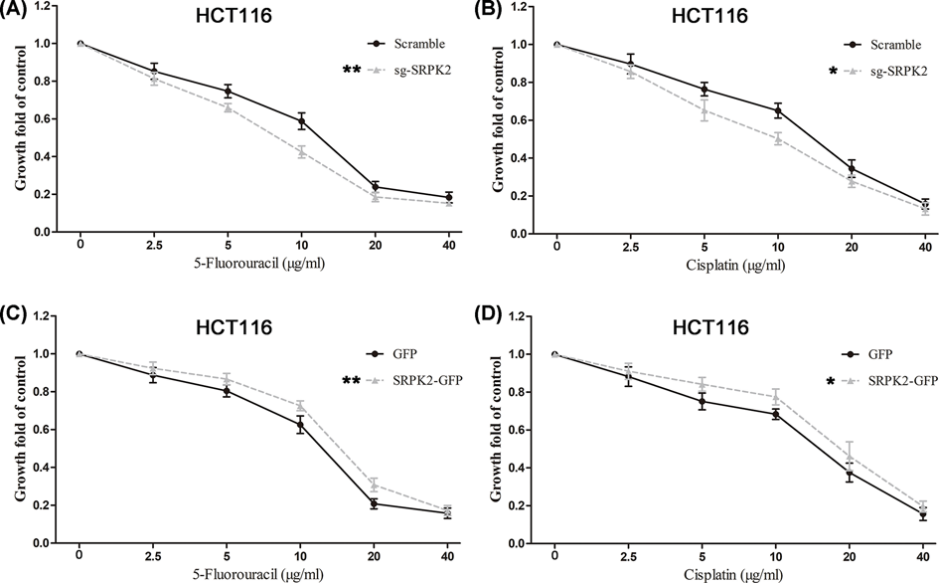
SRPK2 regulates the chemotherapeutic resistance of wtp53 CRC cells (**A** and **B**) SRPK2 silencing significantly decreased the chemotherapeutic resistance to 5-fluorouracil (A) or cisplatin (B) in HCT116 cells. (**C** and **D**) SRPK2 overexpression significantly enhanced the chemotherapeutic resistance to 5-fluorouracil (C) or cisplatin (D) in HCT116 cells. Data are shown as the mean ± SD. Statistical significance was determined by Student’s *t*-test. *P < 0.05, **P < 0.01.

Cell migration and invasion assays showed that SRPK2 silencing significantly inhibited the migration and invasion (Figure 5A,B), whereas SRPK2 overexpression obviously increased the migration and invasion (Figure 5C,D) of HCT116 cells. Moreover, CCK-8 assays revealed that SRPK2 silencing or overexpression significantly enhanced and decreased, respectively, the chemosensitivity to 5-fluorouracil and cisplatin in HCT116 cells (Figure 6).

It is known that wtp53 plays a crucial role in the malignant biology and chemosensitivity of various cancers including CRC [17,18]. Thus, we examined whether SRPK2 regulated cell migration, invasion and chemosensitivity through the wtp53 signaling pathway in CRC cells.

**Figure 7:**
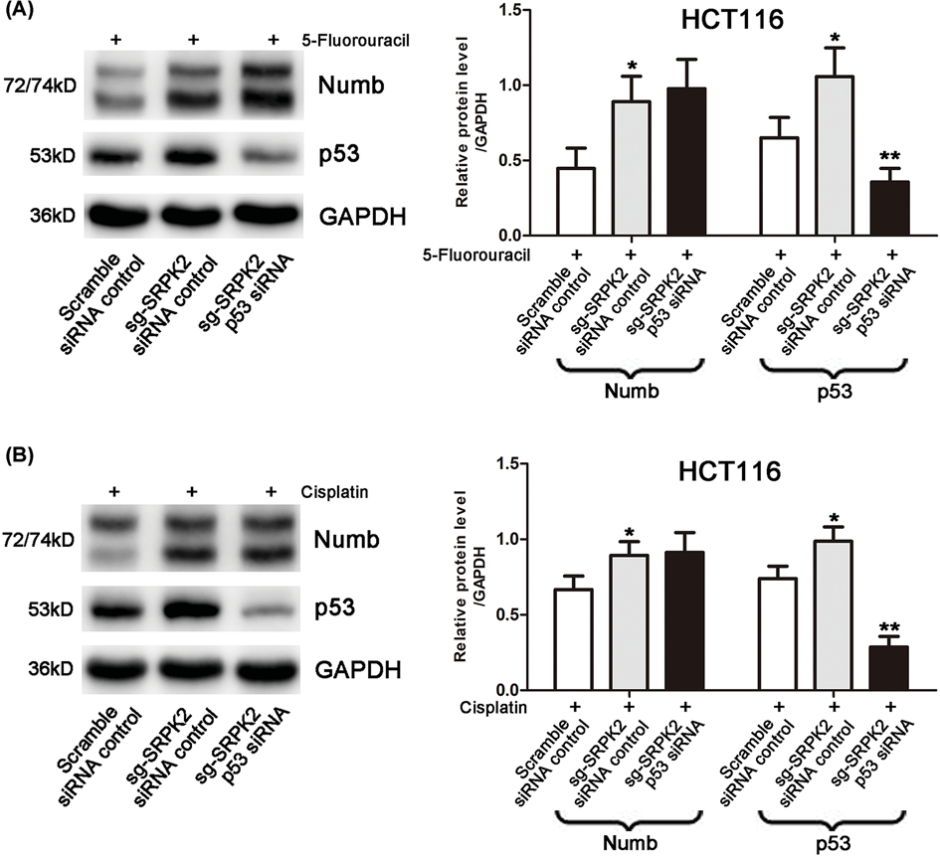
Numb and p53 expression in wtp53 CRC cells with the treatment combinations shown under chemical agent treatment (**A** and **B**) p53 knockdown reversed the up-regulation of wtp53 expression induced by SRPK2 silencing under 5-fluorouracil (A) or cisplatin (B) treatment in HCT116 cells. Data are shown as the mean ± SD. Statistical significance was determined by Student’s *t*-test; *P < 0.05, **P < 0.01.

**Figure 8:**
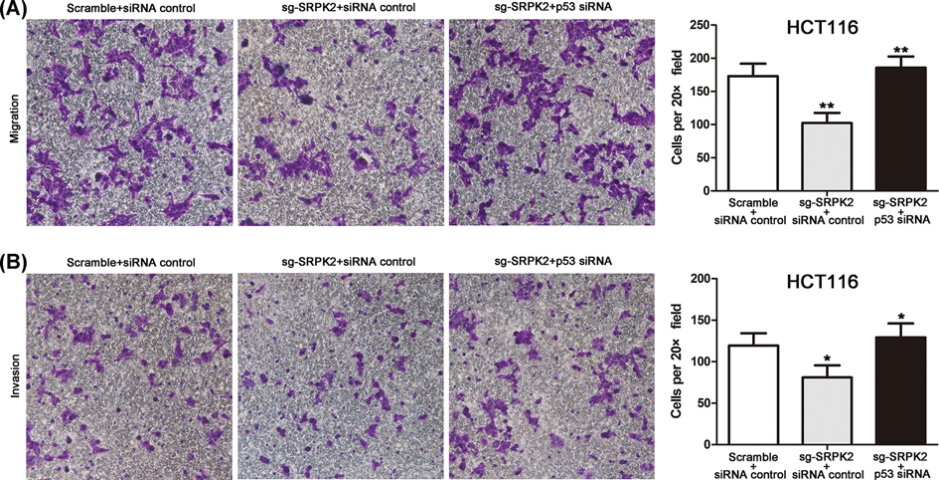
Cell migration and invasion assays in wtp53 CRC cells transfected with the combinations shown (**A** and **B**) p53 knockdown reversed the decrease in cell migration (A) and invasion (B) induced by SRPK2 silencing with 5-fluorouracil treatment in HCT116 cells. Original magnification ×200. Data are shown as the mean ± SD. Statistical significance was determined by Student’s t-test; *P < 0.05, **P < 0.01.

**Figure 9:**
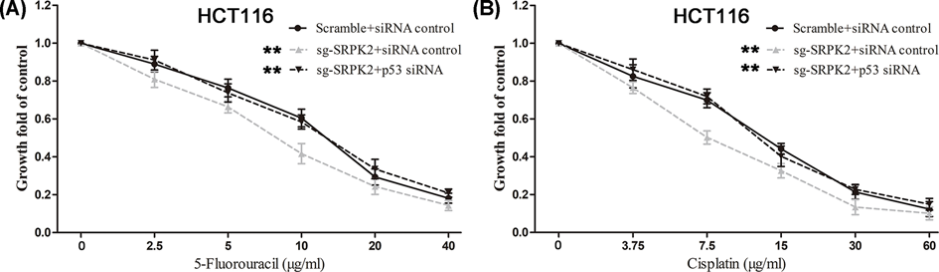
Chemotherapeutic resistance of wtp53 CRC cells treated with chemical agents in the combined protein transfection and expression groups shown (**A** and **B**) p53 knockdown reversed the decreased chemotherapeutic resistance to 5-fluorouracil (A) or cisplatin (B) induced by SRPK2 silencing in HCT116 cells Data are shown as the mean ± SD. Statistical significance was determined by Student’s *t*-test. **P < 0.01.

To induce wtp53 activation, transfected HCT116 cells were first pretreated with IC50 doses of chemical agents. We found that compared with the sg-SRPK2 siRNA control group, p53 expression and the chemosensitivity of the sg-SRPK2 p53 siRNA group were significantly decreased, and cell migration and invasion were significantly increased, which indicated that wtp53 knockdown significantly reversed the effects induced by SRPK2 silencing, including the up-regulation of wtp53 protein (Figure 7), the decrease of cell migration and invasion (Figure 8), and the increase of chemosensitivity to 5-fluorouracil or cisplatin (Figure 9). Taken together, these results suggested that SRPK2 enhanced cell migration and invasion and decreased the chemosensitivity of CRC cells in a p53-dependent manner.

## Discussion

Alternative splicing is a common phenomenon in mammalian cells that is tightly associated with the post-splicing steps of mRNA transcription, as well as the synthesis of various protein isoforms [19,20]. Abnormal patterns of premRNA alternative splicing are prevalent in human malignancies [21,22], yet the molecular mechanisms of these alterations remain poorly defined. The SR proteins are splicing regulators, characterized by a unique domain enriched with serine/arginine repeats, which have essential roles in premRNA alternative splicing and gene expression [23,24]. Phosphorylation of SR proteins has a critical role in the regulation of their activity [25]. Several kinases that phosphorylate SR proteins have been identified, including SR protein kinases (SRPKs) [26,27], Clk/Sty protein kinase [28], DNA topoisomerase I [29] and AKT [30]. Thus, SRPK2, a kinase of the SRPK family, phosphorylates SR proteins and has essential roles in premRNA alternative splicing, which may be linked to human diseases, including cancers, if splicing dysfunctions occur.

In 1998, the cloning of SRPK2 was simultaneously reported in mice and humans [4,5]. SRPK2 is predominately localized in both the cytoplasm and nucleus [31], which was confirmed in our IHC results. Early studies revealed a generalized role of SRPK2 in human hematologic and solid cancers. SRPK2 promoted the cell proliferation of leukemia by regulating cyclin A1 (not cyclin A2) expression [11]. Consistent with the present study, SRPIN340, an inhibitor of SRPKs, showed antileukemic effects [32]. In hepatocellular carcinoma, SRPK2 silencing elevated Numb expression, which decreased cell migration and invasion by down-regulating Akt phosphorylation and c-Myc expression [33]. We recently reported that Numb knockdown decreased chemosensitivity by down-regulating p53 expression in pancreatic cancer [34], and SRPK2 regulated cell migration, invasion and chemosensitivity via the Numb and p53 signaling pathway [15]. Therefore, SRPK2, Numb and p53 may play an important role in the progression of CRC, but this role has not yet been investigated.

In the present study, we found that SRPK2 was elevated in 111 CRC samples, in accordance with the report by Wang et al. [13] of 28 colon cancer tissues. SRPK2 expression was closely associated with multiple aggressive clinicopathologic characteristics of CRC patients, including tumor differentiation, T stage, lymph node metastasis and UICC stage, which was consistent with our previous study in pancreatic cancer [15], showing that SRPK2 expression was positively associated with tumor T stage and UICC stage. Zhuo et al. [13] reported that SRPK2 protein expression was significantly associated with more advanced pathological stage in their own clinical TMA (tissue microarray) cohort and the TCGA (The Cancer Genome Atlas) dataset in prostate cancer. Hence, these findings suggest that SRPK2 promotes the progression of human solid cancers.

Mutations of the p53 gene are frequently detected in human cancers [35]. Activation of mtp53 promotes pro-survival signals and tumorigenesis but not the loss of wtp53 [36,37]. Our previous study showed that SRPK2 had no association with mtp53 in pancreatic cancer tissues but negatively regulated wtp53 protein levels in pancreatic cancer cells under chemical agent stimuli [15]. In CRC, we observed that SRPK2 had no association with mtp53 in SW480 and SW620 cells, but regulated cell invasion, migration and chemosensitivity to 5-fluorouracil or cisplatin by down-regulating Numb and wtp53 expression in HCT116 cells.

Numb is a key determinant of cell fate that was originally discovered in Drosophila [38]. Several reports have shown that Numb acts as an oncogene or tumor suppressor in the development of various human malignancies [39–41]. Generally, Numb contains a proline-rich region (PRR) and a phosphotyrosine binding (PTB) domain [42] that controls the function of p53 by the PTB domain [43]. In wtp53 CRC cells, we found that SRPK2 silencing or overexpression alone negatively regulated Numb protein levels but had no effect on basal wtp53 levels. However, under the treatment of chemical agents, wtp53 was significantly activated. Meanwhile, SRPK2 silencing simultaneously induced an increase in Numb and wtp53 proteins; conversely, SRPK2 overexpression simultaneously decreased Numb and wtp53 proteins. Additionally, these three proteins could be endogenously immunoprecipitated without or with external stress. Moreover, our previous studies showed that SRPK2 down-regulated the level of wtp53 by reducing Numb protein expression, and Numb knockdown significantly reversed the elevation of wtp53 protein induced by silencing SRPK2 [15,34]. Therefore, these results suggest a contributory role for these proteins in the initiation and progression of CRC and that wtp53 is the terminal target protein, a finding that has not been reported in CRC.

Cisplatin is one of the most widely used anticancer agents [44], and 5-fluorouracil is the first-line chemotherapeutic agent for CRC [45]. Unfortunately, chemoresistance severely limits the therapeutic potential of these drugs in CRC. Additionally, strong malignant biology contributes to the unsatisfactory prognosis of CRC. Thus, a better understanding of the molecular mechanisms could facilitate the development of effective biological and pharmacological interventions for CRC. To date, very little attention has been paid to the role of SRPK2 in malignant biology and chemoresistance in CRC. In the present study, we showed that SRPK2 silencing decreased cell invasion and migration and increased chemosensitivity to 5-fluorouracil or cisplatin, all of which could be reversed by p53 knockdown. p53 plays a critical role in the malignant biology and chemosensitivity of various human malignancies [17,18]. We first verified that SRPK2 regulates cell invasion, migration and chemosensitivity of CRC in a p53-dependent manner.

Here, we reveal a previously unknown signaling pathway by which SRPK2 regulates cell migration, invasion and chemosensitivity in CRC. Collectively, our current findings suggest that SRPK2 promotes the development and progression of CRC in a p53-dependent manner. Further work is needed to assess the molecular mechanisms of the crosstalk between SRPK2, Numb and p53 *in vitro* and *in vivo*.

## Abbreviations

CRC: colorectal cancer
FBS: fetal bovine serum
mtp53: mutant p53
PRR: proline-rich region
PTB: phosphotyrosine binding
qRT-PCR: Quantitative real-time reverse-transcription polymerase chain reaction
SRPK2: serine-arginine protein kinase 2
SRPKs: SR protein kinases
SR: serine/arginine
TCGA: The Cancer Genome Atlas
TMA: tissue microarray
wtp53: wild-type p53
